# Supplementary figures and images for: Trypanosoma cruzi PARP is enriched in the nucleolus and is present in a thread connecting nuclei during mitosis
Source: PLoS One. 2022 Dec 30;17(12):e0267329. doi: 10.1371/journal.pone.0267329 (PMC9803098; doi:10.1371/journal.pone.0267329)

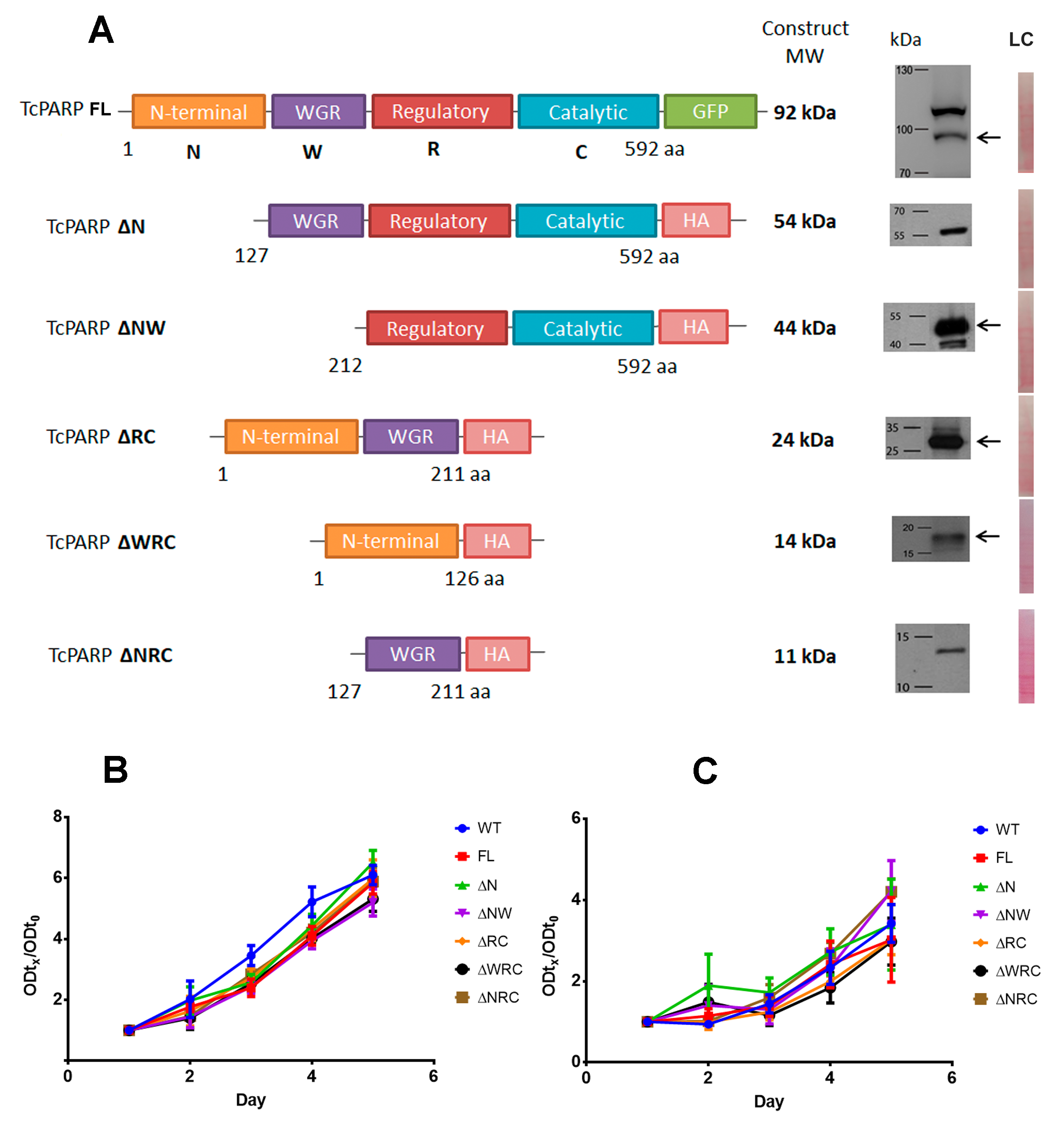

Supplement: S1 Fig — (A) Constructs bearing different combinations of TcPARP domains expressed in transgenic epimastigotes. Left Panel: Schematic Diagram of truncated TcPARP recombinant proteins made through the combination of different domains and fused to HA tag. TcPARP full length was tagged with GFP. Numbers under each diagram indicate amino acid positions. Theoretical molecular weight was calculated considering the molecular weight of HA (1 kDa) or GFP (27 kDa). Right panel: Western Blot on total extract of T. cruzi epimastigotes transfected with plasmids bearing the indicated constructs, using the appropriate antibody against the tag. The arrows indicate the expected molecular weight corresponding to the expression of the different constructs. LC: Ponceau-stained membrane as loading control. Parasite growth curves under basal or oxidative stress condition. Epimastigotes of transgenic and wild type (WT) lines in exponential growth phase were incubated in basal condition (B) or in the presence of 200 μM H2O2 for 10 minutes (C). After treatments cells were collected by centrifugation at 3000g/5min at room temperature, washed with PBS, suspended in LIT (6.106 parasites ml-1) and placed in 96-well plates in 100 μl aliquots. The H2O2 concentration used was sublethal and permitted parasite growth. All the growth curve tests were carried out in three or more independent experiments, each one of them in technical triplicates. Results are expressed as relative growth, OD 600nm at each day was normalized to the initial value (OD tx/OD t0). One-way ANOVA, show no significant differences compared to the wild type. (TIF) [file pone.0267329.s001.tif]

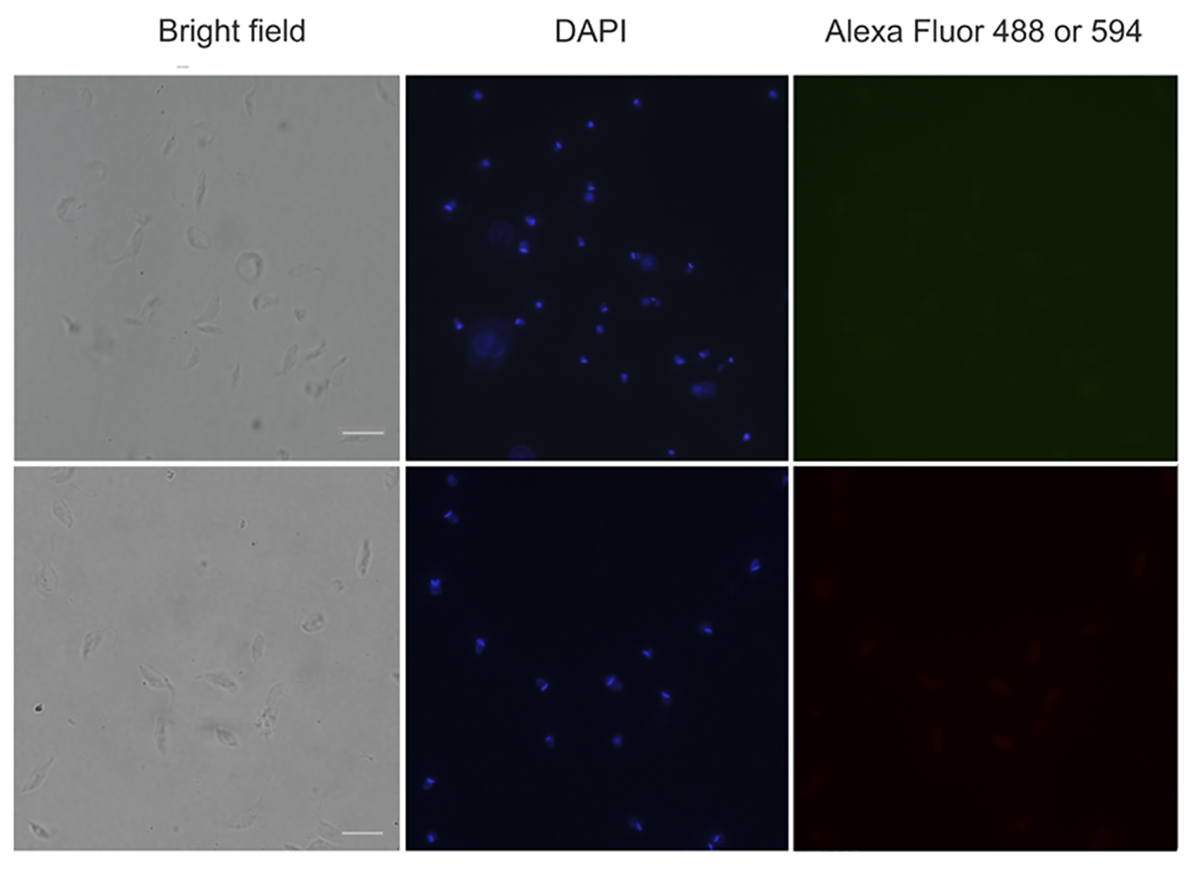

Supplement: S2 Fig — Epimastigotes were incubated without primary antibodies. Alexa Fluor 488 goat anti-rat IgG conjugated antibody 1:500 (upper panel) and Alexa Fluor 594 goat anti-mouse IgG conjugated antibody 1:500 (lower panel) were used as a secondary antibodies. Blue represents DAPI staining of kinetoplast and nuclear DNA. Samples incubation in the absence of the primary antibody showed no unspecific binding of secondary antibodies. Scale bar: 5 μm. (TIF) [file pone.0267329.s002.tif]

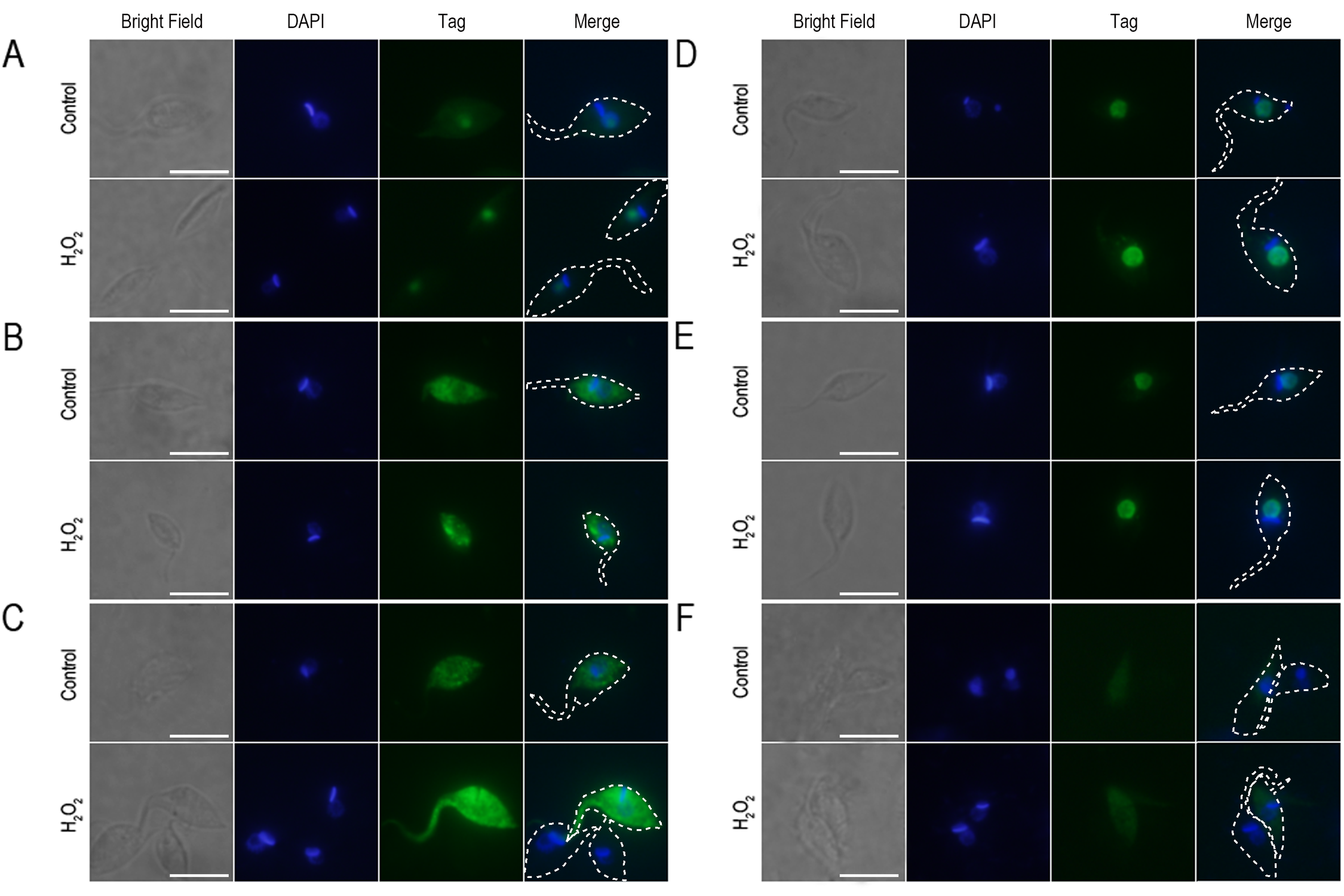

Supplement: S3 Fig — Indirect immunofluorescence of T. cruzi epimastigotes (CL Brener strain) that overexpress TcPARP-FL or different combinations of protein domains, under basal conditions (Control) or treated with 200 μM hydrogen peroxide (H2O2) for 10 minutes. (A) TcPARP-FL. (B) TcPARPΔN. (C) TcPARPΔNW. (D) TcPARPΔRC. (E) TcPARPΔWRC. (F) TcPARPΔNRC. Scale bar: 10 μm. (TIF) [file pone.0267329.s003.tif]

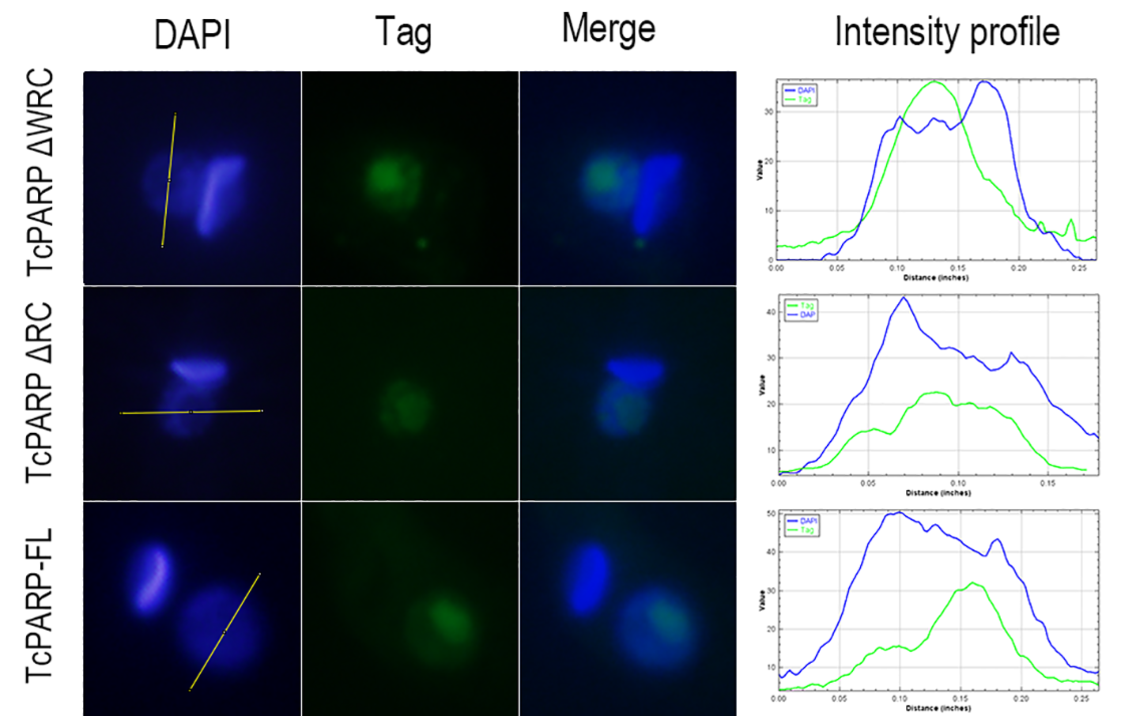

Supplement: S4 Fig — Indirect immunofluorescence of T. cruzi epimastigotes (CL Brener strain) that overexpress TcPARP-FL, TcPARPΔRC or TcPARPΔWRC. Fluorescence intensity profile for each channel (Blue or Green) was analyzed using ImageJ software on the line shown in the DAPI panel. (TIF) [file pone.0267329.s004.tif]

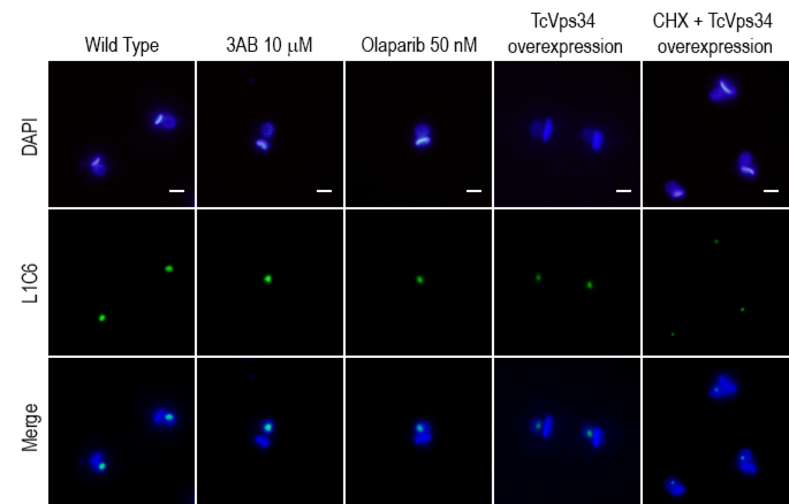

Supplement: S5 Fig — Actively growing wild type epimastigotes preincubated for 1 h in the presence of the NAD+ analogue, 3 aminobenzamide (3AB), or the TcPARP inhibitor, Olaparib; and PI3K TcVps34 overexpressing parasites (TcVps34 overexpression) in the presence or absence of 100 μg.ml-1 cycloheximide (CHX), were fixed and labeled with L1C6 antibody. Scale bar: 5 μm. (TIF) [file pone.0267329.s005.tif]
