# Supplementary material for: Trypanosoma cruzi PARP is enriched in the nucleolus and is present in a thread connecting nuclei during mitosis
Source: PLoS One. 2022 Dec 30;17(12):e0267329. doi: 10.1371/journal.pone.0267329 (PMC9803098; doi:10.1371/journal.pone.0267329)
Supplement: Attachment — Submitted filename: Response to reviewers.pdf [file pone.0267329.s007.pdf]

Response to Reviewers

Dear Editor

We are very grateful for the reviewers' comments and believe they will greatly improve the manuscript. We are sending the manuscript including both the reviewers´ and editor's recommendations.

All material has been deposited in a repository and the FIGSHARE files are the followings:

PAR in the thread DOI 10.6084/m9.figshare.20639706

TcPARP in thread DOI 10.6084/m9.figshare.20639892

TcPARP delta WRC in the nucleolus DOI 10.6084/m9.figshare.20640018

TcPARP-FL in the nucleolus DOI 10.6084/m9.figshare.20639856

WT in the nucleolus DOI 10.6084/m9.figshare.20641164

Colocalization TcPARP, L1C6 DOI 10.6084/m9.figshare.20640792

Colocalization TcPARP constructs, L1C6 DOI 10.6084/m9.figshare.20640837

Supplementary Figure 4 Fig DOI 10.6084/m9.figshare.20640402

Reviewer #1:

I really enjoyed reading the manuscript of Kevorkian et al on PARP in T. cruzi. In my opinion, the authors presented a comprehensive story definitely worth publishing. I have one suggestion and one cosmetic critique.

Suggestion: please extend info on PARP in other trypanosomatids

Cosmetic critique: please fix references for consistency and compliance with PLoS One style (word capitalization)

We would like to thank the referee for the kind comments on our work.

A paragraph with more information about PARP in trypanosomatids has been included in the introduction section (see lines 54-69).

The references format is in compliance with PLoS One style in the revised manuscript.

Reviewer #2:

Kevorkian et al. examin the subcellular localisation of TcPARP in epimastigote stages of the Kinetoplastid parasite Trypanosoma cruzi. TcPARP is the only PARP in these parasites. They generated a panel of overexpression cell lines, each overexpressing a recombinant form of TcPARP, ranging from full length overexpression to various truncated versions of the protein. This work follows on from previous work in this group into PARP functions across several trypanosomatid species.

In summary they show:

1) *N-terminal region of TcPARP is important for the nuclear localisation under DNA damage conditions, despite the authors being unable to find an NLS in TcPARP.*
2) *TcPARP additionally appears co-localize, in some cases, with the site of the nucleolus.*
3) *In mitotic cells, TcPARP additionally formed a 'thread-like' localisation spanning between the two dividing nuclei in dividing cells. This localisation pattern correlates with the locations of tubulin, a nucleolar marker (L1C6), and ADP-ribose polymers (PAR).*

They conclude:
TcPARP may play undiscovered roles (in T. cruzi) relating to nucleolar metabolism and/or spindle function in addition to prior described roles in DNA repair/damage signalling.

Significance:

It has been known for some time that PARPs localise to the nucleolus, with domains situated in the N-terminal region required for DNA binding and nuclear localisation (the N-terminal carries a nuclear localisation signal (NLS) in other Eukaryotes. In this regard, the finding is not new to the PARP field ***but this localisation pattern for TcPARP has not been shown previously in T. cruzi and it is important to consider this may be a key finding for T. cruzi biology****. This work may promote*

further examination of more diverse role for PARylation in this parasite. Particularly as aspects of gene expression regulation, cell cycle control and DNA repair are unorthodox when compared to more 'canonical' Eukaryotes.

We would like to thank reviewer 2 for critical reading of our manuscript and all comments that have helped us to improve the here revised version.
All suggested modifications, as well as the minor orthographic corrections, have been included in the present manuscript.

Major Concerns:

This study is largely descriptive based on (at the face of it) a small number of images.

There is limited quantitative data to support the findings of the authors, just statements made regarding localisation patterns and a low number of cells shown as examples. I feel it would be possible with the IFA images they present to perform some quantification which would help to elevate the paper further. This means it can be unclear at times what the authors are trying to propose. In previous papers they have quantified IF results.

IFA images quantification has now been included in the manuscript and in the figure legend when appropriate.

There is no functional data relating to the localisation of TcPARP to the nucleolus.

I feel this may not be entirely necessarily for this study but would be nice to see. They have a panel of overexpression mutants but there is very minimal characterisation of them despite some appearing to have slightly different growth patterns from the WT cell line (perhaps not significant but may be worth looking at in terms of DNA damage conditions etc)

We made these strains expressing these constructs as a tool to better analyse the location of TcPARP in the nucleus and specially to describe the novel finding of its location in the thread that connects both nuclei in mitosis. We have also evaluated the growth curves of the different transgenic parasites under stress conditions (200 µM H_2O_2) but we did not see significant differences between them. These results therefore seemed unattractive to include in the manuscript and we thought that it would not contribute to in the main point in our manuscript. However, we are sending the editor and reviewer these results (See Figure 1 below) for their consideration.

In the revised manuscript we have clarified which statistical test was used and the number of repetitions performed for the obtention of the results depicted in S1Fig.

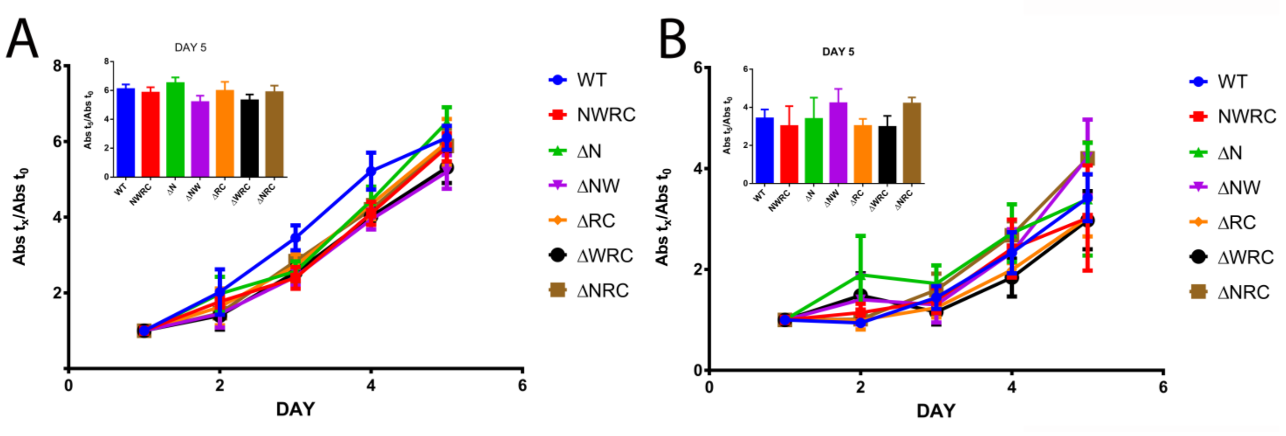

Figure 1. **Effect of the overexpression of TcPARP constructs over parasite growth.** Growth curves of *T. cruzi* (CL Brener strain) epimastigotes under (A) basal conditions (untreated) and (B) 200 µM H_2O_2 treatment of transgenic and Wild Type (WT) lines. Epimastigotes in exponential growth phase were treated with 200 µM H_2O_2 for 10 minutes, washed and resuspended in LIT medium. The H_2O_2 concentration used was sub lethal and permitted parasite growth. The readings were made daily at 600 nm in a microplate reader. Inset: Readings at Day 5 post treatment. Each curve represents a different transgenic epimastigote where absorbance at each day is relativized to absorbance at day 1 vs days of reading.

Error bars show the SEM of five independent experiments. A one-way ANOVA revealed no significant differences between the different growth curves.

Also, why not use the inhibitor to look at spindle formation/cytokinetic defeats etc? Why not carefully profile the spindle progression across mitosis and the formation of the thread structure?

We appreciate the reviewer's interest in our work and we believe that these suggestions could be very valuable for future research. The performance of the suggested experiment presents several challenges: epimastigotes are very small compared to mammalian cells, so it is therefore difficult to get clear images of some cellular structures. Moreover, markers that allow the precise tracking of the different stages of mitosis are not readily available for this model. These facts difficult the possibility to make a precise profile of the cells progression across mitosis, even when working on synchronized cell cultures as we have previously done.

However, we performed a proof of concept with synchronized epimastigotes and observed the mitotic spindle in the G2-M phase of the cycle in parasites incubated both in the absence and in the presence of 100 nM Olaparib, a known PARP inhibitor. We were able to observe that the spindles are formed in both preparations without detecting differences. In any case, we believe that in order to obtain conclusions, better studies are required and further researches are needed. The results can be seen at FIGSHARE DOI 10.6084/m9.figshare.21108895.

Lack of reporting relating to data/experiments

- *Basic descriptions missing relating to the data and methods*
- *How many times an experiment has been performed?*
- *How the data has been processed?*
- *What the error bars represent?*
- *Some controls for IFA images etc?*
- *Type of microscope?*
- *Image processing pipeline?*

- *No section in the methods part that describes for instance cell growth assays (though it is in the figure legend, it should be a method)*
- *No primer sequences have been provided for the generation of the truncated mutants*
- *More references relating to PARP functionality could be included in the intro – i.e to the different functions it performs*
- *Are these experiments only performed using one clone?*

We have paid attention to each item addressed by the reviewer and have entered the corresponding information in the manuscript.
Regarding the last point, we have performed the experiments on antibiotic-selected polyclonal populations for each cell line, as described in Materials and methods section. Specific points are clarified below.

Specific Examples of Data Missing:

SFig. 1A

Western Blots:

- *No loading controls are used – how 'overexpressed' are the proteins relative to a housekeeping control?*

  The gels were made on different days, but the samples were quantified and the same amount of total protein (40 µg) was seeded in each well for all experiments (see Materials and Methods). After transfer, the membranes were checked by Ponceau staining on the same membrane that was used for the WB. Although the reviewer`s point regarding the comparison of expression levels between recombinant structures is interesting (comment below), this comparison is not part of the experimental questions addressed in the current manuscript.

- *What if you are not seeing phenotypes simply due to poor overexpression of one truncated piece Vs another – looks like from the WB that the expression of the recombinant version of TcPARP is different in each cell line.*
  pRIBOTEX vector is not episomally maintained but integrated into a chromosome indistinguishable from the one encoding rRNA. It is a very useful tool since it allows more stable strains but with different degrees of expression, which cannot be easily regulated.
  In this study we were interested in knowing if the parasite strains that express the different constructs had altered their growth capacity with respect to WT, but it was not our intention this time to study differences in other phenotypes of such parasite strains, as was stated before.

- *Is there a negative (untagged) control/ vector control to account for non-specific bands?*
  These controls are always performed, but they were not included in the figures to make them simpler. In any case, all the constructs have different molecular weights, which are reflected in the different patterns that do not present a common band attributable to the vector or something that suggest an unspecific binding of the antibody.

- *What are the other bands on the blot? Several blots have additional bands*
  PARP undergoes several post-translational modifications, of which the most important one is self-PARilation. We thought this is likely the case of TcPARP-FL, which contains all its domains. In this case, the upper band would be proposed for modified TcPARP, as this is a regularly pattern in these WBs. We believe that in the case of delta NW and delta NRC partial degradation or incomplete transcription could have generated bands slightly smaller.

- *I would recommend showing the complete WB uncut given the aforementioned?*
  We believe that for a better figure design the fragment shown is adequate.

- *What ladder has been used?*

We usually used SDS-PAGE - Prestained Protein Ladder – *Blue Plus®* IV Protein Marker (10-180 kDa) Catalog Number：DM131-01- TransGen Biotech Co., LTD. We have incorporated this information to Materials and methods section.

Proliferation curve:

SFig 1B & C

- *Looks like cell line delta-N is growing better than WT cells on a day-to-day basis?*

  The difference in growth rate is not statistically significant, according to figure 1 previously shown, nor did we see a different behaviour in the strain at longer times. Furthermore, we studied the cell doubling time for each strain and we did not find significant differences either (see Figure 2)

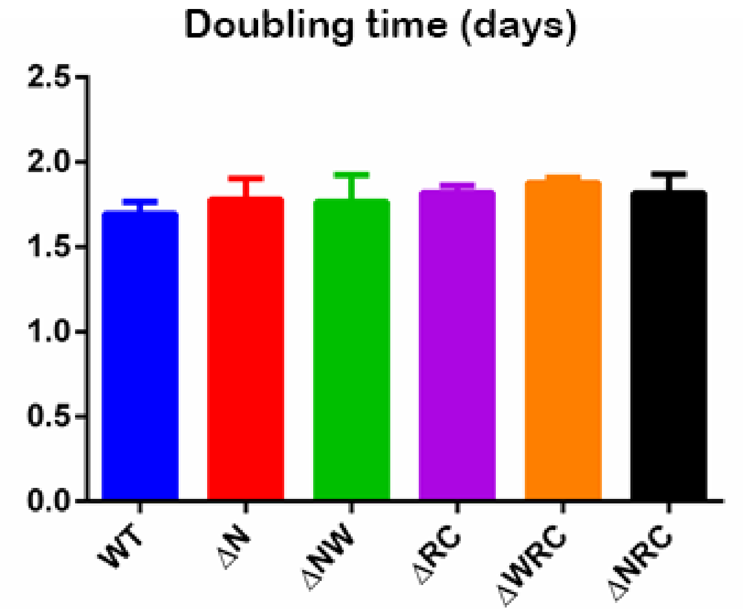

Figure 2: Doubling times of epimastigote cultures during the exponential phase of the growth curve (between day 3 and day 7) (n = 3).

- *Are there any statistics for this data set?*
- *How many times was this experiment performed? There are no details regarding replicates or what the error bars indicate (technical or biological?) SEM?*

All the growth curve tests were carried out in three or more independent experiments, each one in technical triplicates as well. Results are expressed as means +/- SEM. Statistical analysis: Growth curves: Two-way ANOVA; Relative growth on day 4: One-way ANOVA. Results show no significant differences compared to the WT.
We have incorporated this paragraph for clarification in Materials and methods section and in the figure legend.

- *Why are the proliferation and WB referred to in methods and not in the first part of the results section?*
  Our objective was to study the domain responsible for the localization of TcPARP in the nucleus and highlight the interesting finding that TcPARP and the PAR polymer are present in the nucleolus and in the thread in dividing nuclei. The different strains obtained expressing mutants were simply a useful tool for our studies and that is the reason because we decided include these results in Materials and Methods section, since we believe they could be useful for anyone else trying to replicate our experiments or conducting other evaluations.

SFig 2:

- *Untagged control for non-specific antibody staining?*
  Untagged control is showed in figure 1 and 4 upper panels.

- “White Field” – I think this should be “Bright Field”?
  We apologize for the mistake; it has been corrected in all the figures.

- *Number of replicates?*
  All the growth curve tests were carried out in three or more independent experiments, each one of them in technical triplicates. This statement was added to the legend figure.

- *How was the cell body defined (i.e how the white dashed lines were generated)?*

In the Merge quadrants some cells were contoured to illustrate the morphology of the parasites according to the bright field in the corresponding left panel. The dashed-line silhouette was then overlapped on the merge images using the ImageJ tools included in the ROI manager.

- *How were the images processed?*
  Are all the images on the same scale
    - *Each image panel set should have own scale bar.*

  The ImageJ free software was used for image processing.
  We add the corresponding scale bar to the figures.

- *What microscope were the cells captured on?*
  Samples were analyzed using an Olympus BX41 epifluorescence microscope.
  We added this information in Materials and Methods section.

SFig. 3:
I would replace the term overexpressant. “TcVsp34 overexpression”
(See comments above also which apply to this figure)
Figure and legend were changed as suggested.

Figures (Main)
Comments again apply to all figures:

1) *Bright Field?*
   This has been corrected in all figures.

2) *Scale bars – I would put on each Bright Field image as it cannot be assumed they are all on the same scale*
   Scale bars have been added to all figures.

3) *How many times was this experiment performed?*
   a. *Images are representative of?*
4) *How are the images processed?*

Control images without primary antibodies were taken in an analogous condition, during the same microscopy session. All images in each experimental series were taken with the same setting at the same microscope session. If modified, all were subjected to the same degree of brightness/contrast adjustment, including the control without a primary antibody. The ImageJ free software was used for confocal image processing and JaCoP Fiji's plugin was used for colocalization analysis. We have incorporated this clarification in the Materials and methods section. Number of experiment was introduced in the legend figures.

a. *What microscope was used?*
   Samples were analyzed using an Olympus BX41 epifluorescence microscope or a Leica SPE confocal microscope (see Materials and Methods).

5) *Quantification? Co-localisation plots of signal in Image J?*
   The Image J program was used for both fluorescence intensity measurement and confocal microscope image processing. For the co-localization assay, the JaCoP (Image J) tool was used. The signal from two channels (red and green) was analyzed in each single epimastigote.
   Co-location coefficients were incorporated into the text or figure legends when appropriate.

6) *Are all the cells in mitosis at the same stage of mitosis? In some cases for instance the TcPARP delta WRC has a thicker appearance (Fig. 3, 4) vs much thinner in Fig. 2?*
   The cultures were not synchronized, however from the images we assume that the stages analyzed were a late stage in mitosis, as is discussed in the Discussion section.

7) *Some bright field images are missing*
   We have included the bright field only in the first figures to show readers outside the field the epimastigote morphology. Addition of an extra panel with bright field would oblige to reduce other images with loss of information.

TcPARP is enriched in the nucleolus of epimastigotes

Comments:

Line 134: re-localise from where? This needs to be stated

The sentence was clarified.

Line 134: “These” results

The mistake in line 143 was corrected.

Line 135: “different arrangements” you need to define this better – what are you aiming to do – refer to the supplementary figure here and not in the methods.

We agree with the referee's observation, hence supplementary figure S1 is now mentioned in Results section.

Line 145: the use of the word ‘notoriously’ doesn’t fit in the sentence. Do you mean consistently or?

The term notoriously was replaced by "particularly", since we want to highlight this new observation compared to what was previously described for TcPARP localization.

Line 147: this isn’t quantified so how are you making this statement that it is higher fluorescence – it is just an observation by eye?

Also, these are overexpression mutants – are there differences between different truncated cell lines in terms of nucleolar signal intensity associated with PARP? i.e does one mutant show a higher signal expression vs another? (again, ties in with the WB data about overall expression levels of TcPARP)

We added a supplementary figure (S3-Fig) in the revised manuscript showing this observation and a plot of the fluorescence intensity profile for each channel: Blue (DAPI) and Green (Tag). Analysis on the line shown in the DAPI panel was done with ImageJ software.

Lines 157-160:

State again which truncated versions? Only those that have N-terminal sections?

This paragraph was clarified, as suggested.

You are suggesting that the reason you see the mis-localisation is due these cells being in stationary.

- *You want to look at dividing cells but are you collecting cells too far into stationary phase and this is why you are seeing this?*
  The reviewer observation is pertinent. As we stated in the text, we describe this observation only in some cells. Our experiments are performed with cultures on day four after subculturing, which corresponds to an exponential growth stage. Our reference to stationary cells pointed to previous observations by other researchers which we do not believe are the reason for the observation in our case. We change this statement in the text to try to clarify this.

- *Do you see multiple foci as reported by Gluenz? They say they see many foci of the antibody in stationary cells.*
  Even though the staining was not uniform we were not able to define them as Gluenz does in her work.

- *Where are the examples of the cells that have this type of localisation? Do they look like stationary cells? Are they damaged cells? (yH2A staining for genotoxic damage for instance)*
  As was stated before, our redaction was not clear so it was changed to avoid misleading.

- *Is the nucleolar staining found in every cell? you say that that is not the case, and some is found in the nucleoplasm? How many cells in the population?*
  We quantified this observation: only 27 % TcPARPΔWRC overexpressing parasites showed nucleolar localization vs nucleoplasm localization (n=125), whereas 51% of TcPARP-FL parasites showed nucleolar vs nucleoplasm localization (n= 159). This quantification was incorporated to the manuscript.

- *How does this relate to the cell cycle given the nucleolus is consistent across all cell cycle stages? Is there always PARP signal associated with the nucleolus?*
  We have not carried out studies across all cell cycle stages, but we agree with the reviewer that it would be interesting to study it in the future.

Line 165- you mean of your overexpressed construct? I guess stationary phase epis would have reduced transcription relative to log phase.
As indicated before, this statement was rewritten for clarification.

Line 169: this is not quantified so how are you making this statement?
We measured the average area corresponding to the nucleolus for the epimastigotes in the different experimental conditions by using Image J software and found the following results: 0.00194 ± 0.00132 (n=29) for WT, 0.00233± 0.00217 (n=58) for 3AB, 0.00187± 0.00281 (n=32) for Olaparib, 0.00103±0.00149 (n=154) for Vps 34 overexpressors and 0.00059±0.00061 (n=59) for Overexpressors + CXH treated parasites. A high dispersion associated to the data rendered the differences as not statistically significant. We have modified the paragraph accordingly.

Line 178: "Scale bar" not just bar
It was corrected.

TcPARP is present in a connecting wire between nuclei of dividing epimastigotes

Generally: I would choose either 'wire' or 'thread' when referring to the localisation.
Thread was chosen to describe the thin structure that joins the dividing nuclei and the term wire has been removed from the manuscript.

Line 183: I would add more detail here – what nucleolar proteins? This implies you checked the localisation dynamics for several nucleolar proteins and not just L1C6.
We have only localized L1C6 nucleolar marker. The sentence was rewritten for clarification.

Line 186: the cores of what?
The term “cores” was replaced by “nuclei”

Line 185: Again, what nucleolar proteins? L1C6 is but tubulin is not just a nucleolar protein?
The sentence was reformed.

Line 187: You have three versions that carry the N-terminal tag but only two are shown in IFAs with the thread location why? (in figure 2 there are the two) but in figure 3 there are the three?
Relating to the figure:

- *Is it always the case that the truncated version produce a ‘thinner’ thread?*
- *Are these cells at the same stage of mitosis?*
- *Does this happen in every single dividing cell?*
- *Why not include FOV images or many more examples rather than one per cell line?*
- *Legends: Line 195 and 200 – Scale bar*
- *Line 195: corresponding*

In the figures we show a single representative cell because we wanted to show specific details along the manuscript. Given the size of the parasites, a field with many cells would make it difficult. More examples can be found in the repository.
A thinner thread was not always observed for the truncated versions (see Fig 4). Although we agree with the hypothesis that this is dependent on the mitosis stage, as we worked with non-synchronic cultures, identifying a large number of dividing cells was difficult.
Legend and other mistakes were corrected.

PARylated proteins are present in the connecting thread

Line 204 You should elaborate here more about PAR and the assay you will use for non-experts – just a sentence to help.
A sentence was added as suggested and the paragraph is now clearer.

Line 207: How do your over expressors respond to genotoxic stress – more sensitive or less?? Do they produce more PAR (how is this quantified?) Would be good to know if they produce more-or-less relative to WT cells for example.

We have quantified the amount of polymer present in the nuclei of parasites over expressing different constructs by using ImageJ software. A representative image of how the over expressors behave when challenged with H_2O_2 (200 uM) is shown in figure 3 of this text and reviewers can find a table with the processed data. We could not conclude if they produce significant different amount of PAR in oxidative stress condition because of the high dispersion of the data.

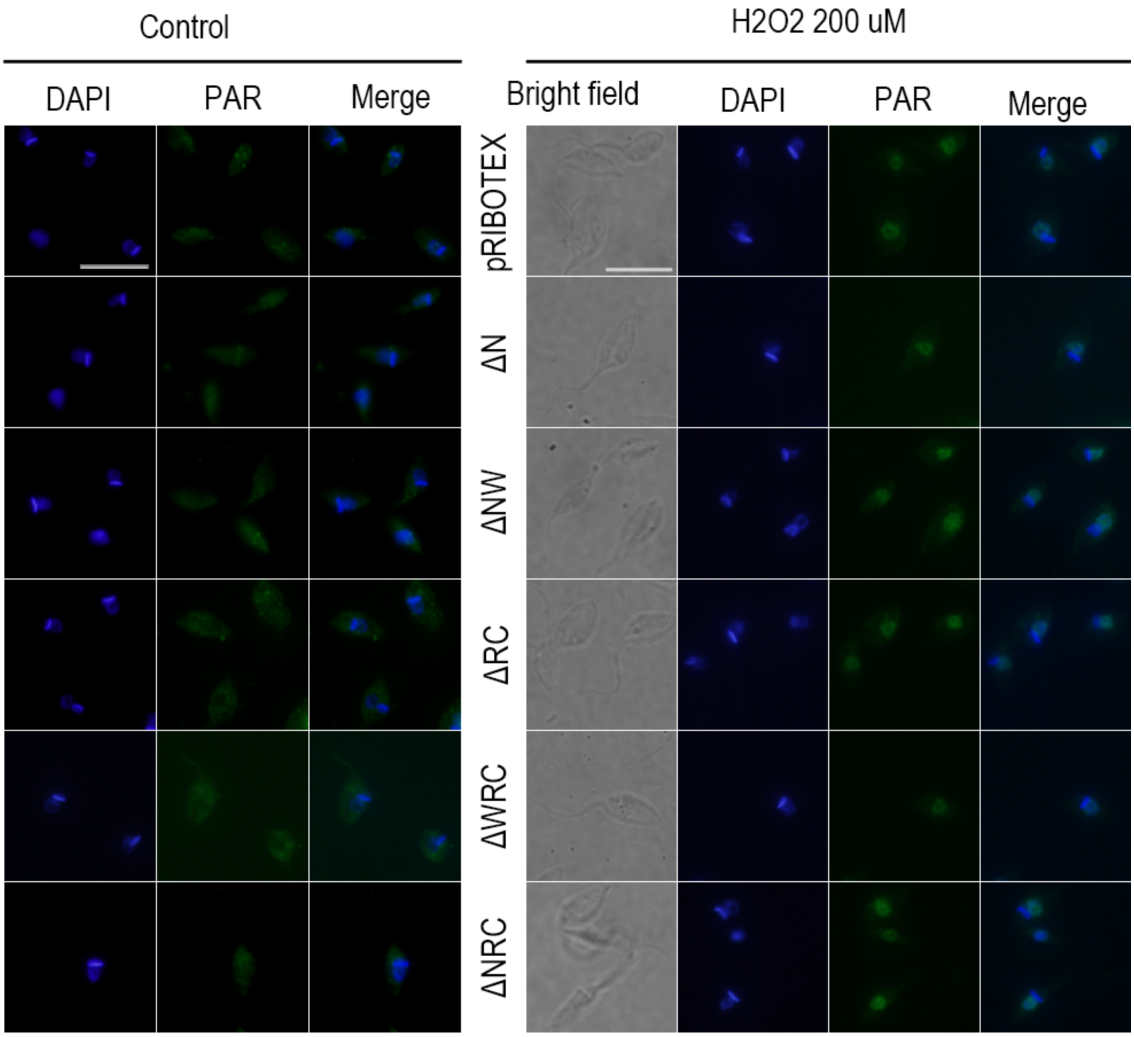

Figure 3. Effect of oxidative stress on PAR level in epimastigotes overexpressing TcPARP domains. IF of transgenic CL Brener epimastigotes under basal conditions

(Control) or after 200µM H_2O_2 treatment for 10 minutes. An antibody that recognizes PAR (green) was used.

Table. Effect of oxidative stress on PAR level in epimastigotes overexpressing TcPARP domains.

	**WT**	**pRibotex**	**ΔWRC**	**ΔNRC**	**ΔRC**	**ΔNW**	**ΔN**	**FL**
**Average**	0,2441	0,1330	0,0856	0,3122	0,0816	0,1501	0,1216	0,2110
**SD**	0,0863	0,0467	0,0713	0,3012	0,0447	0,0665	0,0736	0,1244
**N° Parasites**	10	49	75	47	80	76	88	15

Line 210: dividing 'nuclei'
This was corrected.

Line 212: I think here it would be important to see what happens when you delete or deplete TcPARP and show that this activity is ablated in the thread. Why not use the inhibitor to test this? Is it possible or does the inhibitor disrupt cell cycle progression?
We have previously demonstrated (PloS One 2012) that PARP-specific inhibitors were capable of inhibiting the proliferation of *T. cruzi* epimastigotes in nanomolar concentrations. As it was said before, we believe that it would be very interesting for our future publications to deeply study whether spindle alterations are produced by these inhibitors.

Line 245: why again not investigate this in more detail?
We agree with the reviewer in that it would be interesting to further investigate this phenomenon in the future. However, we consider that it is worth communicating the results already obtained to the research community as they might encourage colleagues working on this topic in trypanosomatids or other parasites.

Lines 250-254:
Are these factors present in T. cruzi?

We have incorporated new information and references related to these proteins that improved the discussion section.

Line 390: space missing
Corrected.

Line 24: molecular switch -> "molecular switches"
Corrected.

Line 29: I would say something like 'thread-like' localisation
Corrected.

Line 31: keep consistent how you describe the localisation – before you say 'thread' here you say 'wire'
All "Wire" were replaced by "Thread" along the manuscript.

Line 43: should this not be 'polymerases' as there is more than one PARP in some eukaryotes?
Corrected.

Line 50: 'discontinued" I'm not not I agree with th
Discontinued DNA was replaced by DNA strand breaks.

Line 56: I would define this better – i.e is the loss associated with different pathologies, like what?
Information on pathologies associated with PARP and PAR was incorporated to the introduction section.

Line 58: specially -> "with particular focus on Trypanosoma cruzi"
Corrected.

I would add a bit more information about Chagas disease to put into context why it is useful to understand Tc biology, especially as it should be written for non-experts
A paragraph was added according to the reviewer´s suggestion.

Line 60: add in the names of the developmental staged and where you find them
This information was introduced as suggested.

Line 62: What are these domains associated with in terms of function – would be good to add a bit of context. – why not see if you can find the domain using Alpha-Fold server for kinetoplastids?
This description was detailed in previous papers cited in the introduction section. However we introduce readers on this topic in the new version of the manuscript.

Line 63: what is important about the N-terminal zinc-finger domain?
This was clarified in the same paragraph.

Line 64: what do you mean by ‘basic’? you mean aa content or? Define
This was corrected.

Line 64: are the functions of hPARP-2 and 3 similar to PARP1 – I would comment on this as you imply here that TcPARP is similar to PARP-1 but you say it actually similar at the N-terminus to PARPs2 and 3. This needs to be a bit clearer described overall in this part.
This was clarified.

Line 66: state where you find it under normal conditions
This information was added.

Line 66: “As it occurs” - > “Akin” PARP
This sentence was corrected as suggested by the reviewer.

Line 78: “co-localize’ - > ‘co-localizes’
This mistake was corrected.

Line 106: “whole cell lysates from transgenic epimastigotes were quantified”
Corrected.

Line 269: are you sure that there is no function for TcPARP in the cytoplasm? It re-localises under DNA damage to the nucleus, but it doesn’t mean that it does absolutely nothing in the cytoplasm?

We do not know if TcPARP has any function in the cytoplasm or in other organelle. But this location of a single PARP in these trypanosomatids would make it possible for it to have other functions beyond those described. Anyway, this paragraph of the discussion was rewritten and is now clearer.
