# Supplementary material for: Trypanosoma cruzi PARP is enriched in the nucleolus and is present in a thread connecting nuclei during mitosis
Source: PLoS One. 2022 Dec 30;17(12):e0267329. doi: 10.1371/journal.pone.0267329 (PMC9803098; doi:10.1371/journal.pone.0267329)
Supplement: Attachment — Submitted filename: Review_comments.docx [file pone.0267329.s006.docx]

Kevorkian *et al.* examin the subcellular localisation of TcPARP in epimastigote stages of the Kinetoplastid parasite *Trypanosoma cruzi*. TcPARP is the only PARP in these parasites. They generated a panel of overexpression cell lines, each overexpressing a recombinant form of TcPARP, ranging from full length overexpression to various truncated versions of the protein. This work follows on from previous work in this group into PARP functions across several trypanosomatid species.

**In summary they show:**

1) N-terminal region of TcPARP is important for the nuclear localisation under DNA damage conditions, despite the authors being unable to find an NLS in TcPARP.
2) TcPARP additionally appears co-localize, in some cases, with the site of the nucleolus.
3) In mitotic cells, TcPARP additionally formed a ‘thread-like’ localisation spanning between the two dividing nuclei in dividing cells. This localisation pattern correlates with the locations of tubulin, a nucleolar marker (L1C6), and ADP-ribose polymers (PAR).

**They conclude:**

TcPARP may play undiscovered roles (in *T. cruzi*) relating to nucleolar metabolism and/or spindle function in addition to prior described roles in DNA repair/damage signalling.

**Significance:**

It has been known for some time that PARPs localise to the nucleolus, with domains situated in the N-terminal region required for DNA binding and nuclear localisation (the N-terminal carries a nuclear localisation signal (NLS) in other Eukaryotes. In this regard, the finding is not new to the PARP field **but this localisation pattern for TcPARP has not been shown previously in *T. cruzi* and it is important to consider this may be a key finding for *T. cruzi* biology**. This work may promote further examination of more diverse role for PARylation in this parasite. Particularly as aspects of gene expression regulation, cell cycle control and DNA repair are unorthodox when compared to more ‘canonical’ Eukaryotes.

**Major Concerns:**

**This study is largely descriptive based on (at the face of it) a small number of images.**

There is limited quantitative data to support the findings of the authors, just statements made regarding localisation patterns and a low number of cells shown as examples. I feel it would be possible with the IFA images they present to perform some quantification which would help to elevate the paper further. This means it can be unclear at times what the authors are trying to propose. In previous papers they have quantified IF results.

**There is no functional data relating to the localisation of TcPARP to the nucleolus.**

I feel this may not be entirely necessarily for this study but would be nice to see. They have a panel of overexpression mutants but there is very minimal characterisation of them despite some appearing to have slightly different growth patterns from the WT cell line (perhaps not significant but may be worth looking at in terms of DNA damage conditions etc)

Also, why not use the inhibitor to look at spindle formation/cytokinetic defeats etc?

Why not carefully profile the spindle progression across mitosis and the formation of the thread structure?

**Lack of reporting relating to data/experiments**

- Basic descriptions missing relating to the data and methods
- How many times an experiment has been performed?
- How the data has been processed?
- What the error bars represent?
- Some controls for IFA images etc?
- Type of microscope?
- Image processing pipeline?
- No section in the methods part that describes for instance cell growth assays (though it is in the figure legend, it should be a method)
- No primer sequences have been provided for the generation of the truncated mutants
- More references relating to PARP functionality could be included in the intro – i.e to the different functions it performs
- Are these experiments only performed using one clone?

**Specific Examples of Data Missing:**

**SFig. 1A**

**Western Blots:**

- No loading controls are used – how ‘overexpressed’ are the proteins relative to a housekeeping control?
- What if you are not seeing phenotypes simply due to poor overexpression of one truncated piece Vs another – looks like from the WB that the expression of the recombinant version of TcPARP is different in each cell line
- Is there a negative (untagged) control/ vector control to account for non-specific bands?
- What are the other bands on the blot? Several blots have additional bands
- I would recommend showing the complete WB uncut given the aforementioned?
- What ladder has been used?

**Proliferation curve:**

**SFig 1B & C**

- Looks like cell line delta-N is growing better than WT cells on a day-to-day basis?
- Are there any statistics for this data set?
- How many times was this experiment performed? There are no details regarding replicates or what the error bars indicate (technical or biological?) SEM?
- Why are the proliferation and WB referred to in methods and not in the first part of the results section?

**SFig 2:**

- Untagged control for non-specific antibody staining?
- “White Field” – I think this should be “Bright Field”?
- Number of replicates?
- How was the cell body defined (i.e how the white dashed lines were generated)?
- How were the images processed?
- Are all the images on the same scale
  - Each image panel set should have own scale bar.
- What microscope were the cells captured on?

**SFig. 3:**

I would replace the term overexpressant. “TcVsp34 overexpression”

(See comments above also which apply to this figure)

**Figures (Main)**

Comments again apply to all figures:

1) Bright Field?
2) Scale bars – I would put on each Bright Field image as it cannot be assumed they are all on the same scale
3) How many times was this experiment performed?
   a. Images are representative of?
4) How are the images processed?
   a. What microscope was used?
5) Quantification? Co-localisation plots of signal in Image J?
6) Are all the cells in mitosis at the same stage of mitosis? In some cases for instance the TcPARP delta WRC has a thicker appearance (Fig. 3, 4) vs much thinner in Fig. 2?
7) Some bright field images are missing

**TcPARP is enriched in the nucleolus of epimastigotes**

**Comments:**

Line 134: re-localise from where? This needs to be stated

Line 134: “These” results

Line 135: “different arrangements” you need to define this better – what are you aiming to do – refer to the supplementary figure here and not in the methods.

Line 145: the use of the word ‘notoriously’ doesn’t fit in the sentence. Do you mean consistently or?

Line 147: this isn’t quantified so how are you making this statement that it is higher fluorescence – it is just an observation by eye?

Also, these are overexpression mutants – are there differences between different truncated cell lines in terms of nucleolar signal intensity associated with PARP? i.e does one mutant show a higher signal expression vs another? (again, ties in with the WB data about overall expression levels of TcPARP)

Lines 157-160:

State again which truncated versions? Only those that have N-terminal sections?

You are suggesting that the reason you see the mis-localisation is due these cells being in stationary.

- You want to look at dividing cells but are you collecting cells too far into stationary phase and this is why you are seeing this?
- Do you see multiple foci as reported by Gluenz? They say they see many foci of the antibody in stationary cells.
- Where are the examples of the cells that have this type of localisation? Do they look like stationary cells? Are they damaged cells? (yH2A staining for genotoxic damage for instance)
- Is the nucleolar staining found in every cell? you say that that is not the case, and some is found in the nucleoplasm? How many cells in the population?
- How does this relate to the cell cycle given the nucleolus is consistent across all cell cycle stages? Is there always PARP signal associated with the nucleolus?

Line 165- you mean of your overexpressed construct? I guess stationary phase epis would have reduced transcription relative to log phase.

Line 169: this is not quantified so how are you making this statement?

Line 178: “Scale bar” not just bar

**TcPARP is present in a connecting wire between nuclei of dividing epimastigotes**

Generally: I would choose either ‘wire’ or ‘thread’ when referring to the localisation.

Line 183: I would add more detail here – what nucleolar proteins? This implies you checked the localisation dynamics for several nucleolar proteins and not just L1C6.

Line 186: the cores of what?

Line 185: Again, what nucleolar proteins? L1C6 is but tubulin is not just a nucleolar protein?

Line 187: You have three versions that carry the N-terminal tag but only two are shown in IFAs with the thread location why? (in figure 2 there are the two) but in figure 3 there are the three?

Relating to the figure:

- Is it always the case that the truncated version produce a ‘thinner’ thread?
- Are these cells at the same stage of mitosis?
- Does this happen in every single dividing cell?
- Why not include FOV images or many more examples rather than one per cell line?
- Legends: Line 195 and 200 – Scale bar
- Line 195: corresponding

**PARylated proteins are present in the connecting thread**

Line 204 You should elaborate here more about PAR and the assay you will use for non-experts – just a sentence to help.

Line 207: How do your over expressors respond to genotoxic stress – more sensitive or less?? Do they produce more PAR (how is this quantified?) Would be good to know if they produce more-or-less relative to WT cells for example.

Line 210: dividing ‘nuclei’

Line 212: I think here it would be important to see what happens when you delete or deplete TcPARP and show that this activity is ablated in the thread. Why not use the inhibitor to test this? Is it possible or does the inhibitor disrupt cell cycle progression?

Line 245: why again not investigate this in more detail?

Lines 250-254:

Are these factors present in *T. cruzi*?

Line 390: space missing

Line 24: molecular switch -> “molecular switches”

Line 29: I would say something like ‘thread-like’ localisation

Line 31: keep consistent how you describe the localisation – before you say ‘thread’ here you say ‘wire’

Line 43: should this not be ‘polymerases’ as there is more than one PARP in some eukaryotes?

Line 50: ‘discontinued” I’m not not I agree with th

Line 56: I would define this better – i.e is the loss associated with different pathologies, like what?

Line 58: specially -> “with particular focus on Trypanosoma cruzi”

I would add a bit more information about Chagas disease to put into context why it is useful to understand Tc biology, especially as it should be written for non-experts

Line 60: add in the names of the developmental staged and where you find them

Line 62: What are these domains associated with in terms of function – would be good to add a bit of context. – why not see if you can find the domain using Alpha-Fold server for kinetoplastids?

Line 63: what is important about the N-terminal zinc-finger domain?

Line 64: what do you mean by ‘basic’? you mean aa content or? Define

Line 64: are the functions of hPARP-2 and 3 similar to PARP1 – I would comment on this as you imply here that TcPARP is similar to PARP-1 but you say it actually similar at the N-terminus to PARPs2 and 3. This needs to be a bit clearer described overall in this part.

Line 66: state where you find it under normal conditions

Line 66: “As it occurs” - > “Akin” PARP

Line 78: “co-localize’ - > ‘co-localizes’

Line 106: “whole cell lysates from transgenic epimastigotes were quantified”

Line 269: are you sure that there is no function for TcPARP in the cytoplasm? It re-localises under DNA damage to the nucleus, but it doesn’t mean that it does absolutely nothing in the cytoplasm?
